# Supplementary material for: Are carbon emissions trading and green financial instruments synergistic? -Comprehensive quantitative research based on content analysis
Source: PLoS One. 2024 Mar 7;19(3):e0298601. doi: 10.1371/journal.pone.0298601 (PMC10919601; doi:10.1371/journal.pone.0298601)
Supplement: S1 Data — (ZIP) [file pone.0298601.s001.zip › Data/地级市Z_F工作报告.zip!/╔╜╢1⁄2/═■║ú╩╨/└┤╫╘╣1⁄2╓┌║┼í╛┬φ┐╦╩2╛▌═°í┐_2012.docx]

各位代表：

现在，我代表威海市人民政府向大会报告工作，请予审议，并请各位政协委员和其他列席人员提出意见。

一、第十五届政府工作回顾

2008年本届政府组成以来，在省委、省政府和市委的坚强领导下，市政府和全市人民一道，突出科学发展主题，克服国际金融危机不利影响，抓住山东半岛蓝色经济区建设战略机遇，加快转变经济发展方式，保持经济平稳较快发展，促进社会和谐稳定，改革开放和现代化建设取得可喜成就。

――稳增长、提质量取得新成效。全市生产总值由2007年的1375.5亿元提高到2011年的2111亿元，年均增长12%；地方财政收入达到136.4亿元，年均增长13.6%；税收占地方财政收入的比重提高4.35个百分点，经济发展的质量效益进一步提升。

――转方式、调结构取得新突破。三次产业比重由9.3：58.2：32.5调整为8.1:54:37.9；新口径高新技术产业产值比重达到33.6%，高于全省6.3个百分点；万元生产总值能耗降低19.9%，主要污染物排放量继续下降，转型升级步伐进一步加快。

――惠民生、促和谐取得新进步。城镇居民人均可支配收入达到2.5万元，农民人均纯收入达到1.2万元，年均分别增长11.6%和12.4%；居民消费价格涨幅低于全国1.1个百分点，人民生活进一步改善。

四年来，主要做了以下工作：

（一）强化民生保障，公共服务水平不断提高。财政民生投入占预算支出的比重达到52%，年均增长29%。扶持4127人创业，转移农村劳动力8.6万人，新增城镇就业20.6万人，城镇登记失业率控制在1.6%以内。实施新型农村和城镇居民养老保险制度，城乡居民适龄人口养老保险参保率达到97.2%。城镇职工医保、城镇居民医保和新农合的最高支付限额分别提高66.7%、30%和166.7%。政府办基层医疗机构全面实施基本药物制度，基本药物价格平均下降45.7%。社会救助体系不断完善，城乡低保标准分别提高20%和65%，荣获“中国爱心城市”称号。促进各类教育协调发展，实施校舍、校园、校车安全工程，强化素质教育，被评为“全国义务教育均衡发展工作先进地区”。中心城区77个城中村实现整体拆迁改造。新建改造农村住房19.1万户。提供经济适用房7480套、公共租赁房3736套、廉租住房556套，发放廉租住房租赁补贴1809户,城镇保障性住房覆盖率达到19.7%。

（二）强化产业培植，转型升级步伐不断加快。制定实施《蓝色经济区发展规划》，加快建设海洋经济六大基地，统筹发展涉海产业，造船完工量达到143.8万载重吨，增长3.9倍；港口吞吐量达到5501万吨，年均增长14.7%；渔业经济总产值达到718亿元，年均增长8.7%。大力发展高效生态安全农业，打造“威海刺参”等特色品牌，新增农产品地理标志品牌19个，粮食产量连续四年稳定在100万吨以上，被评为出口食品农产品质量安全示范市。实施八大产业调整振兴规划，规划建设十大高端产业聚集园区，连续四年开展“自主创新年”活动，建立13个产学研战略联盟，创建8家国家级、75家省级企业创新平台；获得国家科技进步奖6项，专利申请量和授权量年均分别增长24%和29.9%；新增中国驰名商标14件，三角、威高、金猴3家企业获得省长质量奖；规模以上工业总产值达到5248亿元，年均增长14.8%。实施旅游文化产业发展行动，旅游总收入年均增长18%，打响“千里海岸线，一幅山水画――走遍四海，还是威海”的旅游形象品牌；引进永旺、乐天、银座、苏宁、天虹等大型商贸企业，社会消费品零售总额年均增长19.5%，服务业增加值年均增长14.4%，高于生产总值增幅2.4个百分点。实施保护中小企业发展行动，采取促进银企合作、设立科技支行、成立小额贷款公司、引进金融机构等方式，帮助企业化解融资难题，银行贷款余额年均增长17%。5家企业实现上市。个体私营企业由7.4万户增加到11.1万户。主营业务收入过10亿元的工业企业由51家增加到76家，过50亿元的由2家增加到6家。三角、威高、家家悦3家企业主营业务收入突破100亿元。缴纳税收过亿元的企业由11家增加到23家，威高集团突破10亿元。

（三）强化改革开放，经济发展活力不断增强。深化行政审批制度改革，调整取消行政收费项目263个，争取降低工商业用电价格，切实为企业减负让利。推行财政支出绩效评价制度。调整市属重点国有企业和行政事业单位国有资产管理体制。推进集体林权和农村土地承包经营权流转制度改革。开展城市精细化管理行动，出台支持小城镇建设、城市管理体制创新等意见，完善城市社区、公交运营等管理体制。强化产业招商和项目建设，引进美敦力、史丹利、法液空、希斯庄明、华润、雨润、神飞、中航等国内外知名企业，实际到帐外资23.6亿美元，固定资产投资年均增长22.3%。创建船舶、海产品两个国家级出口基地，外贸进出口总额达到169.2亿美元，年均增长12.1%。

（四）强化生态建设，资源环境保护不断加强。健全土地、海域、海岸线、山体等资源的管理机制，推动各类资源统筹保护利用。实施城乡河道、饮用水水源地综合整治工程，建立农村垃圾集中处理机制，建成56处镇级、23处村级污水处理设施，城市环境考核保持全省第一。实施干线公路和乡村道路绿化工程，成片造林40万亩，森林覆盖率达到40%，成功创建国家森林城市。集中治理道路破损、停车秩序、散流物体运输、建筑垃圾、工地扬尘、机动车尾气、违章建筑等市民关心的问题，完成800个村的环境综合整治任务，被确定为全省农村环境连片整治示范市。

（五）强化区域统筹，城乡建设水平不断提升。中心城区启动“两城两区”建设，威海工业新区快速崛起，石岛管理区健康发展，南海新区成为山东半岛蓝色经济区的重点海洋经济新区。高区科技新城、经区临港产业园、环翠区五渚河流域生态休闲度假区、双岛湾生态科技新城加快建设。实施新型城镇化建设行动，荣成市、文登市、乳山市和各中心镇的发展水平全面提升，城镇化率达到58.5%。青烟威荣城际铁路、石岛湾核电站、胶东调水列入国家重点工程，完成358座大中型水库除险加固工程。建成长会口跨海大桥，改造升级干线公路480公里、农村公路990公里。在全国率先开通中韩陆海联运业务，新增万吨级以上泊位11个，威海港新港泊位等级达到10万吨。建成机场国际航站楼，新开通7条航线，旅客吞吐量由60万人次增加到93万人次。铁路客运量由219万人次增加到309万人次。中心城区实施宝泉广场、金线顶、环翠楼公园、江家寨立交桥等重点工程，新建改造城市道路90条，整治河道17条，文化艺术中心等公益设施投入使用，衡量城市市政公用设施水平的主要指标均居全省前列。

（六）强化社会建设，各项社会事业不断进步。加强和创新社会管理，深入推进“平安威海”建设，健全安全生产监管制度、食品药品质量安全管理体制、社会矛盾纠纷排查化解机制和应急管理体系。实施五大文化惠民工程，刘公岛成为国家级文化产业示范基地，农村文化大院建设入选国家公共文化服务体系示范项目，被评为“全国文化体制改革先进地区”。成功举办国际人居节、霍比帆船世界锦标赛、长距离铁人三项世界杯赛、接收台湾长鬃山羊和梅花鹿等重要活动。人口计生、妇女儿童、民族宗教、广播电视、外事、侨务、对台、档案、史志、气象和防震减灾等工作取得新成绩，完成对口援川、援青等帮扶任务，国防动员、民兵预备役和优抚安置等工作进一步加强，被评为“全国人民防空先进城市”，连续六次获得“全国双拥模范城”称号。

各位代表！在推动科学发展、促进社会和谐的同时，我们坚持依法行政，不断强化政府自身建设。实施市县镇政府机构改革，加快转变政府职能。自觉接受人大的法律监督、政协的民主监督和群众的社会监督，办理人大代表建议和政协委员提案878件，办复率达到100%。强化目标绩效管理考核，完善电子监察网络，健全责任追究机制，机关作风有新转变。推进政务公开，加强社情民意调查，倾听群众呼声，竭力为群众办实事、解难题，在全省科学发展综合考核群众满意度调查中，连续四年位居第一。

各位代表！过去四年的成绩来之不易，我们深切地感受到每一份成果都凝聚着全市人民的心血和汗水。在此，我代表市政府，向全市人民，向各民主党派、工商联、人民团体和各界人士，向驻威人民解放军、武警官兵，向各位老领导、老同志，向所有关心支持威海发展的海内外朋友，致以崇高的敬意和衷心的感谢！

在总结成绩的同时，我们也清醒地看到，我市经济社会发展中不平衡、不协调、不可持续的问题依然存在：发展速度和质量效益相对偏低，财税实力和综合竞争力还不够强；产业结构不够合理，海洋产业的深度广度挖掘不够，现代服务业发展相对滞后，新兴产业发展有待突破；外资来源不够多元化，招商引资规模和质量有待提高；大企业数量少，中小企业活力不足，实体经济的基础还不够坚实；城市基础设施承载能力还不够强；交通、人才等瓶颈制约还比较突出；县域经济发展步伐不够快；民生领域的一些矛盾和问题尚未有效解决；政府管理和服务水平有待提高，部分政府工作人员工作标准不高，安于现状、不思进取、小进则满的观念有待转变，敷衍塞责、推诿扯皮、以权谋私等不正之风还不同程度地存在。我们一定正视矛盾和问题，采取更加有力的措施，努力把各项工作做得更好，不辜负人民群众的期望！

二、今后五年政府工作的总体设想

各位代表！今后五年是我市推进科学发展的重要时期。综观国际国内形势，世界经济复苏进程艰难曲折，国际金融危机还在发展，国内经济下行压力依然很大，区域经济竞争日益激烈。但我国发展仍处于大有可为的重要战略机遇期，工业化、城镇化和农业现代化快速推进，山东半岛蓝色经济区上升为国家战略，都为我市今后发展提供了有利条件。我们必须抓住机遇，迎难而上，在应对挑战、化解危机、破解难题中开创科学发展的新局面。

按照市十四次党代会绘就的宏伟蓝图，结合“十二五”规划，今后五年政府工作的总体要求是：高举中国特色社会主义伟大旗帜，以邓小平理论和“三个代表”重要思想为指导，深入贯彻落实科学发展观，强化危机意识，树立赶超思想，激发发展活力，提高发展质量，坚持生态立市、科教兴市、产业强市，坚持民生为重、改革推动、开放引领，着力推进转方式、调结构，促进扩内需、稳增长，统筹城乡发展，壮大县域经济，促进城市文明和谐，在建设现代化幸福威海的道路上迈出坚实的步伐。

今后五年经济社会发展的主要预期目标是：生产总值力争突破4000亿元，年均增长11%；地方财政收入翻番，年均增长15%；城镇居民人均可支配收入和农民人均纯收入年均增长11%；城镇化率力争达到65%，规模速度与质量效益同步快速增长，区域生活品质、城乡文明程度、社会管理水平、生态环境质量明显提升。

（一）坚持产业强市。围绕蓝色经济区建设，以提高质量效益为中心，加快培育更具特色、更具竞争力的产业体系，三次产业比重调整为4.5：52：43.5，促进三次产业在更高层次上协调发展。强化蓝色产业的优势地位。坚持陆海统筹，大力发展现代渔业、船舶修造、港口物流、海产品精深加工产业，培育海洋生物、海洋新能源、海洋高端装备制造等高技术产业，进一步打响“威海海珍品”系列品牌，建成北方重要的海洋食品、药品、保健品等精深加工基地和交易集散中心，将国家船舶出口基地建设成为船舶工业科技创新基地。强化农业的基础地位。落实渔业、果业、畜牧业三大产业振兴规划，加强品牌建设和质量安全管理，提高农业科技成果转化率，大力发展高效生态安全农业，促进农业提质增效、农民持续增收。强化工业的主体地位。坚持传统产业改造与新兴产业培育“双轮驱动”，推进工业化和信息化深度融合，促进新信息、新能源、新材料、新医药、高端装备制造产业加快发展，打造机械制造、电子信息、食品医药、纺织服装4个主营业务收入过千亿元的产业集群。巩固壮大骨干企业，新培育主营业务收入过50亿元的工业企业14家、过100亿元的7家。强化服务业的战略地位。坚持扩大总量与优化结构并举，生产性服务业与生活性服务业并重，鼓励发展服务业新型业态，重点发展金融、会展、文化、旅游休闲、商贸物流、商务服务产业，突破发展软件及服务外包产业，促进现代服务业和先进制造业良性互动。加快建设滨海休闲旅游度假目的地，新增8家4A级以上景区、6家五星级酒店。中心城区建成宝泉广场、高新商贸广场、乐天世纪广场、九龙城等重点工程，商业面积超过150万平方米。开工建设华润中心、绿轴商务金融中心。引进培育带动力强的服务业大项目，扶持重点服务业企业快速发展。

（二）坚持科教兴市。大力实施科教兴市和人才强市战略，推动经济走上创新驱动、内生增长的发展轨道。强化创新驱动。更加注重激发企业的创新活力，引导企业加大研发投入，全社会研发经费投入占生产总值比重达到2.2%，创新型城市建设取得新突破。更加注重发挥创新服务体系的作用，加快公共服务平台和孵化器建设，依托企业再创建50家省级以上创新平台，建成山东船舶技术研究院。更加注重科技成果转化应用，强化产学研合作，高新技术产业产值比重力争每年提高1个百分点。深入实施知识产权战略，加强知识产权信息服务平台建设，制订专利扶持政策，专利申请量和授权量明显提升。强化教育基础。合理配置教育资源，促进各类教育全面发展。深入实施素质教育，促进义务教育优质均衡发展。健全政府主导的学前教育办园体制。推动职业教育与产业发展相结合，根据产业需要调整专业设置和培养方式。加强与山大、哈工大校地共建，支持山东交通学院海运学院异地扩建，促进威海职业学院突出特色、内涵发展，加快建设威海海洋职业学院，提高各类高职院校办学水平。推进民办教育规范发展。强化人才支撑。全力破解制约威海发展的人才瓶颈问题，全面落实人才引进和奖励政策，营造人才创业发展的良好环境，统筹抓好专业技术人才、经营管理人才、实用技能型人才队伍建设。建设高校毕业生创业孵化基地。

（三）坚持生态立市。牢固树立绿色发展理念，倡导低碳增长模式和消费方式，大力推进生态文明建设，建成国家生态市，进一步打响“蓝色休闲之都、世界宜居城市”的品牌。实施最严格的规划控制制度。设立“产业红线”，任何项目都要以环保约束为条件，严格控制“两高一资”和产能过剩行业投资扩张，决不以牺牲环境为代价换取一时的发展。实施最严格的总量控制制度。持续降低能耗水平和污染排放总量，化学需氧量、氨氮、二氧化硫、氮氧化物排放量分别下降6%、8%、13%和15.5%。全面消除劣五类水体。将PM2.5纳入常规空气质量监测体系，空气质量优良率保持在96%以上。森林覆盖率达到42%以上，中心城区建成区绿化覆盖率达到48.5%。实施最严格的资源管理制度。坚持严格保护、规范管理、集约利用的导向，进一步健全自然资源管理体制，力争使每一寸土地、每一段岸线、每一片海域、每一块林地、每一条河流都有人管、管得住、管得好。

（四）坚持改革开放。用改革的办法破解发展难题，用开放的思路拓展发展空间，增强发展的动力和活力。推进重点领域和关键环节改革。深化行政管理体制改革，规范再造服务流程。加强市场化政府投融资平台建设，建立地方政府债务规模控制和风险预警机制，提高城市经营水平。加强公共资源交易的统一规范管理。深化农村综合改革、户籍管理制度改革，推动农村人口向城镇有序转移。实施更加积极的开放战略。把招商引资和项目建设作为经济工作的重中之重，更加注重优化结构和提高质量，以更大的力度推进项目引进和建设，切实提高投资强度。深入实施科技兴贸、以质取胜和市场多元化战略，加快转变外贸发展方式。实施“走出去”战略，加强区域合作，提升开放型经济水平。

（五）坚持区域带动。强化规划引导，统筹城乡、区域协调发展，推动城镇化由注重规模速度向注重内涵质量转变。完善空间布局，加快推进新型城镇化。按照“中心崛起、两轴支撑、环海发展、一体化布局”的思路，做大做强中心城区，培育带动区域发展的龙头；以中心城区为枢纽，以东西拓展、向南延伸为两轴，集中培育两条产业隆起带；沿千公里海岸线，将中心城区、三市城区、沿海城镇串连成线，形成组团发展的城镇群，构建一体发展新格局。推进城市现代化，促进信息技术在城市管理、社会管理、政府管理等领域的应用，构建精细化城市管理格局。统筹区域发展，做大做强县域经济。中心城区加快建设双岛湾生态科技新城，适时启动城市东部新区规划建设。环翠区、高区、经区要提升现代服务业发展水平，打造相对集中的金融、商务、商贸聚集区和科技服务区。荣成、文登、乳山既要注重外延扩张，更要注重内涵发展，勇于赶超先进，壮大特色产业，提高县域经济综合竞争力。强化园区建设，增强产业承载和聚集功能。推动高区、经区深化“二次创业”，实现提速增效；力争出口加工区升级为国家级综合保税区；强力推进工业新区建设，打造先进制造业发展的重要板块；扶持南海新区尽快崛起，促进生态、生产、生活共融，建设现代化生态科技新城，培植威海发展的新增长极；引导各类园区突出特色，打造支撑有力的开放窗口、创新高地、项目聚集区。统筹基础设施建设，破除发展的瓶颈制约。完成青烟威荣城际铁路建设，推进桃威铁路资产重组。实施荣威、文莱、烟海高速乳山段等高速大通道项目，抓好重点港口疏港公路建设，全线贯通滨海旅游景观公路，基本完成“九纵三横一环”干线公路建设。科学确定港口发展定位，强化资源整合与战略合作，抓好威海新港、石岛港、南海新港建设，大力发展临港物流园区。推动机场改扩建，优化航线航班网络。围绕城际铁路威海站和威海北站建成两大综合性交通枢纽。规划论证中心城区轨道交通体系。实施泊于水库、智能电网、供排水、燃气设施等建设改造工程，打造更加完善的基础设施保障体系。

（六）坚持民生为重。始终把改善民生作为政府工作的出发点和落脚点，构建普惠型公共财政体系，提高民生投入占财政支出比重，切实提升公共服务能力，促进基本公共服务均等化。实施积极的就业政策，扩大各项社会保险覆盖面，完善城乡社会保险制度衔接办法，实现各项社会保险跨区域、跨险种转移接续，努力让人人享有充分的社会保障。健全以居家养老为基础、社区服务为依托、机构养老为补充的社会养老服务体系，努力让老年人得到更多关爱。深化医药卫生体制改革，完善覆盖城乡的基层医疗网络和公共卫生服务体系，努力让群众看病更方便、负担更轻。构建更加合理的住房保障体系，建设保障性住房1.8万套，努力让更多的低收入群体住有所居。推动公共设施向农村延伸、公共服务向农村覆盖，建立农村公共服务设施长效管护机制，加强农村住房建设和危房改造，提高农村饮用水质量，实施1152个村的环境整治工程，全面完成农村环境综合整治任务，让农村居民享受更多的均等化服务。全面完成中心城区1500万平方米的老旧居民住宅节能改造。完善社区基本公共服务体系，加强城乡社区文化体育活动场所建设，努力让群众享受更加便利的综合服务。坚持公交优先发展，新增节能环保型公交车300辆，优化公交场站布局，提高建成区公交线网覆盖率，努力让居民出行更加方便。加强和创新社会管理，完善公共安全体系，强化安全生产管理，加强食品药品安全监管，优化社会治安环境，化解社会矛盾，促进社会和谐，努力让群众更有安全感。及时了解和回应不同群体的利益诉求，做好困难群体和低保边缘家庭的帮扶工作，努力让人民群众的生活年年都有新改善，一年更比一年好。

（七）坚持文化繁荣。以文化为城市灵魂、文明为城市追求，加强社会主义核心价值体系建设，深化文明城市、文明行业、乡村文明行动等群众性精神文明创建活动，倡树文明新风，努力争创全国文明城市。进一步健全公共文化服务体系，实现农村文化大院和城市社区文化中心的全覆盖，让群众享受更多文化福利。发展壮大文化产业，培育文化市场，打造一批优势文化产业基地，培植一批龙头文化企业。坚持传承传统文化与弘扬时代精神相统一，保护历史文化遗产，强化城市品牌营销，加强对外文化交流，彰显城市文化风采，培育特色城市文化，提升文化软实力。

各位代表！人民政府的一切权力都是人民赋予的，我们一定牢记责任，不辱使命，坚持依法行政，依靠法律和制度的力量，规范政府行为，增强行政效能，提高服务水平；坚持解放思想，改革创新，切实增强危机感和紧迫感，以只争朝夕的精神，谋划新发展，寻求新突破；坚持逆向切入，从研究解决实际问题入手开展工作，勇于担当，敢于碰硬，不回避矛盾，不掩饰问题，不畏惧困难；坚持求真务实，真抓实干，始终保持敢为人先的闯劲、攻坚克难的韧劲、为民造福的干劲，以脚踏实地的工作，创造出经得起群众考问、经得起历史检验的业绩。

三、2012年政府工作安排

各位代表！今年是新一届政府的起步年。我们一定认清当前复杂严峻的经济形势，做好应对更大困难的准备，充分利用各种有利条件，紧紧围绕主题主线，全面贯彻“稳中求进”的工作总基调，坚定不移扩大内需，大力发展实体经济，加快推动改革创新，竭尽全力改善民生，扎实推进各项工作，努力保持经济平稳较快发展，促进社会和谐稳定。

2012年经济社会发展的主要预期目标是：生产总值增长11%，地方财政收入增长15%，固定资产投资增长18%，社会消费品零售总额增长16%，外贸进出口增长10%，实际利用外资和内资稳定增长，城镇居民人均可支配收入和农民人均纯收入增长11%，居民消费价格涨幅控制在4%左右，城镇登记失业率控制在2%以内，人口自然增长率控制在1‰以内，万元生产总值能耗下降3.7%，化学需氧量、氨氮排放量均下降2.5%，二氧化硫、氮氧化物排放量均下降3%。

（一）突出战略重点，加快推进蓝色经济区建设

落实蓝色经济区发展规划。做好《山东半岛蓝色经济区改革发展试点工作方案》涉及我市的5大领域65项具体工作，设立蓝色经济区建设专项资金，推进国家海产品质检中心、海洋商品国际交易中心和山东船舶技术研究院建设，完善各项配套设施，促进各类要素集聚。

加强蓝色经济园区建设。落实加快推进南海新区跨越发展的政策，调整园区规划，找准产业定位，为尽快实现突破打好基础。推进荣成好运角旅游度假区建设。做好省级海洋特色经济园区和大乳山国家海洋公园的申报工作。推进威海港战略合作和规划建设，威海港国际客运中心投入使用；扩大中韩陆海联运业务，提高口岸通关效率，港口货物吞吐量增长12%。

推进蓝色产业发展。制订养殖水域滩涂规划，出台促进水产种苗业健康发展的意见，扶持30家龙头企业、100家成长型渔业企业发展，提高海产品精深加工能力。加强渔船管理，争取已申报的141艘远洋渔船尽快获批。支持船舶配套企业发展，加强高附加值船型开发，帮助企业化解市场风险。强化海洋科技开发，规划荣成海洋生物产业园，推进传统临海、涉海产业改造升级。

（二）突出创新驱动，调整优化产业结构

发展高效生态安全农业。强化科技兴农，实施新型农民科技培训工程，完善基层农业技术推广网络，实施农业良种工程，建好国家级现代农业示范区。切实落实惠农政策，保障农产品有效供给。实施新一轮“菜篮子”工程，推动农超对接，新建超市直供基地3万亩。发展休闲观光农业，培育特色农产品品牌。推行规模化、标准化养殖，加快现代畜牧业示范区建设。推进农田水利基本建设，发展农业节水灌溉工程13万亩。支持农业龙头企业、农民专业合作组织发展，提高农业的产业化、组织化水平。

调整振兴工业经济。健全经济运行监测调控机制，及时帮助企业解决实际困难，保障工业生产经营稳定，全年工业增加值增长11%。研究制订促进工业转型升级和第二轮扶持企业做大做强的政策，推动骨干企业、重点行业突破发展，增强中小企业抵御市场风险能力。健全煤电油运保障机制，切实保证市场供应。强化战略合作，促进优势企业与国际国内知名企业攀亲结缘，引进战略投资、先进技术、关键设备、优势品牌和优秀人才，培植行业领军企业，提高产业集中度。强化科技创新支撑，全年完成新产品开发1500项。强化项目带动，加强综合协调服务，加大技术改造力度，抓好100个重点工业投资项目，年内完成投资430亿元。强化优势产业集群培植，支持发展关联配套企业，拉长产业链条。实施电子商务“十百千”计划，以信息技术提升传统产业。实施质量振兴、品牌带动战略，新增省级以上品牌26个。

提升服务业发展水平。落实促进服务业发展的40条政策和各项推进机制，抓好环翠区全省服务业综合改革试点区建设。引导企业剥离非核心业务。鼓励发展各类中介服务机构。大力发展家庭和农村服务业。落实房地产市场调控政策，促进房地产业健康发展。推进“万村千乡”市场工程，加快建设社区连锁超市和便民服务设施，全市新增商业面积97.6万平方米。办好国际食品博览会、国际渔具博览会、海峡两岸海洋食品展销会等展会。大力发展旅游休闲产业，加强特色旅游产品开发，实施11个旅游重点项目；推进旅游信息化建设，启动旅游公共服务中心建设；强化政策支持，做好全国旅游标准化试点城市工作，加强旅游市场综合管理，优化旅游整体环境，全年旅游总收入增长15%。

各位代表！科技和人才是产业优化升级的基础。今年我市连续第五年开展“自主创新年”活动，重点强化企业的创新主体地位。支持产学研合作，创建全国蓝火计划试点城市和中国高校威海技术转移中心，启动企业产学研战略合作特聘专家资助计划，全年签署实质性产学研合作项目60个以上。加强科技创新平台、科技中介服务机构和孵化器建设，创建8个省级以上创新平台。推动科技与金融结合，扩大科技支行试点，启动知识产权质押试点工作，支持科技型中小企业发展。引导企业积极申报高新技术企业，用好用活研发费用加计抵扣等优惠政策，提高企业科技经费支出占销售收入的比重。加大知识产权保护力度，全年专利申请量突破4800件。组织实施国外引智项目和人才培养计划，为各类人才创造更加优越的环境，让各类创新要素加快向企业集聚、加快与产业结合。

（三）突出招商引资，提升开放型经济水平

创新招商引资机制。调整充实专业招商机构，提升专业招商队伍水平。坚持内资、外资并重，建立重点项目协调推进机制，组织好20个境内外重点招商活动，优化外资来源结构。充分发挥企业的招商主体作用，强化以商招商、定向招商、配套招商和集群式招商。依据产业规划确定招商引资导向，制订制造业和服务业招商指南，健全招商选资综合评价体系，优化资金投向。创新利用外资方式，力争在引进战略投资、推进外资并购、加大增资扩股、推动境外上市等方面实现新突破。优化招商服务，健全更加周到的全过程服务机制。完善招商引资考核评价机制，更加注重考核真实投资强度，提高招商引资实效。各市区、经济园区是招商引资的主要力量，必须结合各自产业实际强力推进，力争招商项目规模和质量都取得新突破。

强化重点项目建设。健全跟踪服务和督查机制，落实项目推进责任制，对重点项目实行闭环全过程服务，确保招商引资项目及时落地，各类在建项目加快推进、尽快投产见效。今年实施100个市级重点项目，年计划投资318亿元，主要集中在高新技术产业、先进制造业、现代服务业和基础设施等领域。认真落实促进民间投资的一系列政策措施，鼓励各类企业多上项目、上好项目，促进投资规模稳定增长、投资结构持续优化。

转变外贸发展方式。大力实施市场多元化战略，扩大出口信用保险覆盖范围，推进跨境人民币业务，组织好重点经贸展销活动，巩固扩大出口市场份额。抓好国家级出口基地建设和省级出口基地创建工作。调整优化贸易结构，扩大高技术、高附加值产品出口，提升一般贸易比重，提高加工贸易档次和水平。扩大先进技术、关键设备和短缺资源进口。支持优势企业“走出去”，拓展发展空间。

各位代表！对外开放是威海的出路所在，加强招商引资和项目建设是现阶段发展十分关键的大事，必须不遗余力地加快推进。我们要全力强化这项工作，切实调动各方面的积极性，打好招商攻坚战，为威海的发展注入新的强劲动力。

（四）突出机制创新，深化重点领域改革

健全财税管理体制。牢固树立健康财政的理念，进一步提高公共财政管理水平。深化社会综合治税，加大信息管税力度，保证财税总量稳定增长、财税结构稳步优化。深化预算制度改革，完善财政支出绩效评价机制，提高资金使用效率。

完善金融服务体系。健全银企对接长效机制，引导金融机构加大支持实体经济力度，健全服务小微型企业和“三农”的体制机制，力争年末信贷增长超过13%。加强中小企业信用担保体系建设。推进农村信用环境和支付环境建设，全面推行银行卡助农取款业务。加强金融风险控制，确保金融稳定。有效利用资本市场，积极培育上市资源，引进风险投资和创业投资基金，鼓励企业利用债券、票据、私募基金等方式多渠道融通资金。

加强国有资产监管。分领域组建规范国有资产经营公司，探索经营城市的有效途径。加强行政事业单位合同管理，完善资产公开处置规程，切实规范公共资产管理。健全企业国有资产出资人制度，完善经营业绩考核机制，加强对国有企业签约履约行为的监督，确保国有资产保值增值。

深化价格改革。落实价格调控政策，健全价格监测预警和信息反馈机制，强化市场价格监管，保持价格总水平基本稳定。深化资源性产品价格改革，疏导部分公用事业产品价格矛盾。完善社会救助和保障标准与物价上涨挂钩联动机制。

强化医药卫生体制改革。加强医疗保障、医疗服务、公共卫生、药品供应及监管体制等领域的综合改革，扩大基本药物制度实施范围，推进基层医疗机构综合改革，开展公立医院改革试点，实行医药分开、管办分开，破除以药补医机制，加快形成人民群众“病有所医”的制度保障。

深化农村改革。加强农村集体资源、资产、资金管理，做好农村集体产权制度改革试点工作。稳定完善农村基本经营制度，积极推进农村土地承包经营权流转，依法保障农民土地权益。

推进事业单位分类改革。继续做好事业单位清理规范工作，制订完善机构编制、财政管理、人事制度等配套政策。进一步理顺部门职责关系，探索政府购买服务的机制，加快转变政府职能。

优化全民创业环境。全面落实促进非公有制经济发展的两个36条，出台支持小微型企业健康发展的意见，实施扶持中小企业“四项计划”，打好支持各类企业发展的“组合拳”。调整现行涉企收费项目，进一步减轻企业负担。强化创业服务平台建设，加快培育市级小企业创业基地。落实创业扶持政策，调动和保护大学生、归国留学人员、个体工商业主等创业活跃群体的积极性，激发全社会的创造活力，让更多的人在威海实现创业梦想。

（五）突出生态建设，严格保护资源环境

加强资源集约节约利用。完善资源有偿使用制度，健全海岸线控制和集中集约用海制度，严格控制填海项目，严厉查处违法用地、违法取水、破坏山体、非法采砂、私搭乱建等行为。加强耕地保护和节约用地，依法解决批而未供、供而不建问题。健全资源回收利用网络，提高中水回用率，促进循环经济发展。

大力推进节能减排。严守“产业红线”，严格落实节能减排目标责任制。加大节能技术改造力度，加强工业、建筑、交通、公共机构等领域用能管理，完成省下达的淘汰落后产能计划。持续推进工程减排、结构减排和管理减排，实施污水处理厂建设改造工程、燃煤电厂脱硫脱硝工程、燃煤锅炉和燃油机动车改用天然气工程；强化机动车尾气治理，加快淘汰“黄标车”；加强自动化环境安全防控平台建设，确保主要污染物排放量持续削减。

优化生态环境。加强城乡水源地保护，实施重要水体生态环境综合治理，抓好塘坝整治、河道治理和沿海防潮堤工程建设，构建更加安全的水生态环境。规范处置有害固体废弃物，做好土壤污染治理与修复试点工作。强化工业垃圾和餐厨垃圾回收处置管理。加强湿地保护，抓好水土流失区、破损山体及海域海岛海岸带的生态修复，推进荒山、水系、道路绿化，提高森林防火和病虫害防控能力，成片造林7.5万亩。

各位代表！优良的生态环境是威海的宝贵财富。坚持环境优先、生态立市，是全市人民的共识。我们必须更加重视节约资源、保护环境，不给子孙留遗憾，不给自己留骂名，努力使威海的天更蓝、水更清、空气更清新、环境更优美。

（六）突出城乡统筹，加快新型城镇化建设

加强城乡规划建设管理。做好城乡统筹规划、产业园区配套设施规划、中心城区建筑形象引导规划的编制工作。中心城区组织实施30项城建重点工程，宝泉广场、环翠楼公园、江家寨立交桥等8项重点工程主体完工；研究出台城市精细化管理标准细则和考评办法，推行网格化城市管理模式。提升次中心城市的规划建设管理水平，协调推进省级综合配套改革试点、省级经济发达镇行政管理体制改革试点和市级扩权强镇试点工作。

大力发展公用事业。健全公用事业单位的融资机制、运营管理机制和政府支持机制，不断提升基础保障能力。加强地下空间规划利用，搞好城市路网和公共停车场建设，中心城区修建城市道路30条，逐步缓解交通拥堵和停车难问题。加大对城市公交的财政支持力度，更新30辆节能环保型公交车，增加公交线路和营运车辆，改善群众乘车环境。抓好燃气热力配套管网建设，完成供热计量和建筑节能改造600万平方米。加快推进12项输变电工程建设，完善城乡电网结构。

开展和谐城乡建设行动。扎实推进新农村建设，加快建设农村社区服务中心，做好农村规模化集中供水工程试点工作，改善农民生产生活条件。开展“农村公路管理养护年”活动，新建和改造511公里连村路。完成20项国省干线公路的升级改造工程。深化农村环境连片整治示范工作，完成365个村的环境综合整治工程。开展城市环境专项治理行动，加大对户外广告、室外摊点、违章建筑、工地扬尘、噪音污染等方面的整治力度。加强物业管理，规范房屋租赁行为。推进旧居民区综合治理，改善居住环境。

（七）突出文化建设，推进文化繁荣发展

提高城市文明程度。推进社会主义核心价值体系建设，加强社会公德、职业道德、家庭美德教育，做好青少年思想道德教育工作。围绕《全国文明城市测评体系》，加强基础工作，开展争创全国文明城市活动。推动学雷锋、志愿服务活动社会化。大力推进政务诚信、商务诚信、社会诚信建设，健全社会征信系统。

完善公共文化服务体系。实施文化惠民工程，加强基层文化设施建设，规划建设威海图书馆新馆、群众艺术馆新馆。推动公共文化设施免费或优惠向社会开放。加大对各类公共文化活动的支持力度，积极开展群众性文化活动。推动全民健身活动深入开展。促进新闻出版、广播电视事业健康发展。加强文艺精品的创作生产，做好“十艺节”相关筹备工作。加强“老洋房”等历史文化资源的保护。

大力振兴文化产业。完善扶持文化产业的政策体系，推进文化与相关产业互动融合，支持文化创意、影视制作、演艺、动漫等产业发展。加大文化产业招商引资力度，强化文化产业园区、文化产业基地等平台建设，引进培植骨干文化企业，激发文化产业发展活力。举办亚太霍比帆船锦标赛暨黄海国际帆船赛、国际铁人三项赛、国际青少年网球巡回赛，促进体育产业快速发展。

深化文化体制改革。深入实施文化市场规范化建设工程，健全文化市场综合管理机制。按时完成一般性国有文艺院团转企改制任务，培育合格市场主体。创新公共文化设施管理运行机制，推进公益性文化单位内部制度改革，提高公共文化服务效能。

（八）突出改善民生，促进社会和谐稳定

积极扩大就业。加强就业服务和就业援助，做好高校毕业生就业工作，帮助农民工、城镇就业困难群体和退役人员就业，新增城镇就业3万人，转移农村劳动力1万人。组织实施各类就业培训项目，推进素质就业。继续提高企业最低工资标准和工资指导基准线。积极构建和谐劳动关系，改善企业用工环境，加强对劳务派遣的规范管理，妥善处理劳动争议，坚决依法查处拖欠职工工资、侵犯劳动者合法权益的行为。

健全社会保障体系。继续推进各类社会保险扩面提标，提高新农合和城镇居民医保的补助标准，提高大病医疗保障水平，城镇职工养老保险、医疗保险、工伤保险参保人员分别新增1万人。加强公益性养老服务机构建设，建立财政保障的高龄补贴制度。继续增加企业退休人员基本养老金，平均增长10%以上。建立城乡低保和农村“五保”保障标准自然增长机制。推进残疾人社会保障和服务体系建设。做好扶贫开发工作。

办好人民满意教育。全市教育支出占财政支出的比重达到18.5%。实施学生素质提高工程，建设市中小学生综合实践基地，积极培育体育、艺术、科技等特色学校，加强学生心理健康教育。坚持政府主导，扎实推进学前教育三年行动计划，公办及公办性质幼儿园比例提高到55%，扶持普惠性民办幼儿园发展。新建4个中等职业教育市级示范实训基地。加强校园安全工作，在全省提前一年全面完成校舍安全工程，推进校车国家改革试点，探索建立校车运营新模式，筑牢学生的平安防线。

加快发展医药卫生事业。提高基层医疗卫生机构服务能力，加强镇村和社区卫生服务机构标准化建设。扶持中医药事业发展。规范医疗机构诊疗行为，加强医疗技术准入管理工作，诊所规范化建设率达到85%。强化医德医风建设，提高医疗服务水平。完善第三方调解机制，构建和谐医患关系。

抓好保障性住房建设。健全保障性住房政策体系，完善住房保障准入退出机制，扩大住房公积金制度保障范围。重视解决特殊困难群体的住房问题，针对不同收入群体的需求，提供不同层次的保障性住房，年内提供经济适用房600套，建设公共租赁房3000套，努力使低收入家庭住得上房、租得起房。

加强和创新社会管理。深入推进“平安威海”建设，加强社会管理综合治理，依法严厉打击各类违法犯罪活动。探索建立新型社区管理体制，强化社区基础设施服务功能。扎实开展“安全生产基层基础强化年”活动，强化属地管理和行业管理责任，全面落实企业主体责任，坚决遏制重特大事故发生。健全食品药品安全综合协调机制，推行信用档案制度，强化环节监管，开展专项整治，提高抽检率，严肃查处食品安全事件。完善应急管理体系，提高突发事件应急处置和救援能力。充分发挥信访、仲裁、法律服务和法律援助机构的作用，加强人民调解、行政调解与司法调解的衔接联动，妥善化解社会矛盾，保障群众合法权益。

统筹发展各项社会事业。完善计划生育利益导向政策体系，稳定低生育水平，提高人口素质。抓好双拥共建和优抚安置工作，加强国防动员、民兵预备役、人民防空和打击走私等工作。做好民族宗教、妇女儿童、外事、侨务、对台、档案、史志、气象、防震减灾和对口支援工作。

各位代表！惠民生、促和谐，是发展的根本目的，更是政府的职责所在。我们一定牢固树立宗旨意识，贴近群众的需要来谋划工作，倾听群众的呼声来改进工作，尊重群众的意愿来推动工作，多做暖人心、得民心的实事，使政府工作更加符合人民群众的要求，符合人民群众的期待！

四、加强政府自身建设

各位代表！建设法治政府、服务型政府和廉洁政府，是做好各项工作的基础和保障。各级政府和全体政府工作人员要对人民群众抱有敬畏和感恩之心，切实增强责任意识和服务意识，按照“转作风、重规范、提效能”的要求，扎实开展作风建设年活动，加快推进制度建设，促进政府职能转变，努力建设行为规范、务实高效、清正廉洁的人民满意政府。

（一）依法行政，努力打造法治政府。牢固树立法制观念，扎实开展“行政程序年”活动，进一步理顺政府的议事程序和办事规则，促进政府运转更加规范有序。健全科学民主决策机制，严格执行重大决策听证、重要事项公示、重点情况通报等制度，加强行政决策的合法性审查、风险评估和跟踪问效。完善政务公开制度，扩大群众参与度，保障人民群众的知情权、参与权和监督权，提高政府工作透明度。严格规范行政执法程序，按照职权法定、程序合法的要求，依法公开部门职责、执法依据和办事程序，细化量化行政处罚自由裁量权，减少行政执法的随意性。健全督查督办制度，完善目标绩效管理考核体系，加强行政效能监察和责任追究，健全“决必行、行必果”的工作落实机制。做好行政复议委员会试点工作，建立健全独立于执法部门之外的投诉受理机制，让企业和群众受了委屈有地方说理，让滥用权力者得到应有的惩处。

（二）为民务实，努力打造服务型政府。坚持问政于民、问需于民、问计于民，始终从群众利益出发谋划推动工作。充分利用市长公开电话、市长信箱、行风热线、社情民意调查等互动平台，高度重视网络舆情，广泛了解社情民意，努力做到民有所求、政有所应。深化行政审批制度改革，进一步清理审批事项，减少审批环节，压缩审批时限，推行审批流程再造，提高审批效率。加强电子政务建设，完善网上审批系统，扩大网上办事范围。在重点社区和村居设立便民服务点，方便群众办事。加强各级政务服务中心建设，全面理顺办事流程，尽量避免企业和群众“两头跑”。切实转变作风，以治庸强素质，以治懒提效能，以治散正风气，严肃整治行政审批和行政服务中的不正之风，让企业和群众不托人也能好办事、快办事、办成事。

（三）从严治政，努力打造廉洁政府。坚决执行市委的决策部署，依法接受人大的法律监督和政协的民主监督，虚心听取各民主党派、工商联和人民团体的意见，自觉接受社会监督，加强审计、监察等内部监督，让行政权力始终在阳光下运行。加强政府廉政建设，严格执行廉洁自律各项规定，健全惩治和预防腐败体系，强化电子监察等科技防腐手段，深入推进廉政风险防范管理工作，加强对权力运行的制约和监督，促进政风行风建设。坚持勤俭节约，严格控制会议、节庆活动经费支出，严格控制“三公”经费的规模和比例。大力推进专项治理，严肃查处各类违纪违法案件，坚决纠正损害群众利益的不正之风，打造风清气正的政务环境，以良好的形象赢得人民群众的信任和支持。

各位代表！人民政府以人民为重，人民政府为人民服务。加快建设法治政府、服务型政府和廉洁政府，是政府对全市人民的庄严承诺。我们将忠诚履职、积极践诺，不断提高施政能力和服务水平，努力建设有所作为、群众信赖、经得起考验的政府，给全市人民交上一份满意的答卷！让我们紧密团结在以胡锦涛同志为总书记的党中央周围，在省委、省政府和市委的坚强领导下，凝心聚力干事创业，务实奋进转型跨越，为加快建设现代化幸福威海而努力奋斗！
